# Role of 5 wt.% Mg Alloying in Al on Corrosion Characteristics of Al-Mg Coating Deposited by Plasma Arc Thermal Spray Process

**DOI:** 10.3390/ma16083088

**Published:** 2023-04-13

**Authors:** Hwa-Rang Jeong, Jitendra Kumar Singh

**Affiliations:** 1 Department of Architecture Engineering, Daegu Catholic University, 13-13 Hayang-ro, Hayang-eup, Gyeongsan-si 38430, Gyeongsangbuk-do, Republic of Korea; hrjeong@cu.ac.kr; 2 Innovative Durable Building and Infrastructure Research Center, Center for Creative Convergence Education, Hanyang University (ERICA Campus), 1271 Sa-3-dong, Sangnok-gu, Ansan 15588, Republic of Korea; 3 Department of Chemistry, Graphic Era Deemed to be University, Bell Road, Clement Town, Dehradun 248002, Uttarakhand, India

**Keywords:** coating, corrosion, plasma arc thermal spray, electrochemical impedance spectroscopy, scanning electron microscope, X-ray diffraction

## Abstract

The corrosion of steel structures in coastal areas is a major issue. Therefore, in the present study, the protection against the corrosion of structural steel is carried out by depositing 100 μm thick Al and Al-5 Mg coatings using a plasma arc thermal spray process, immersing them in 3.5 wt.% NaCl solution for 41 days (d). To deposit such metals, one of the best known processes, arc thermal spray, is frequently used, but this process has severe defects and porosity. Thus, to minimize the porosity and defects of arc thermal spray, a plasma arc thermal spray process is developed. In this process, we used normal gas to create plasma instead of argon (Ar) and nitrogen (N_2_) with hydrogen (H) and helium (He). Al-5 Mg alloy coating exhibited uniform and dense morphology, where it reduced more than four times the porosity compared to Al, where Mg fills the voids of the coating, resulting in greater bond adhesion and hydrophobicity. The open circuit potential (OCP) of both coatings exhibited electropositive values due to the formation of native oxide in Al, while in the case of Al-5 Mg, the coating is dense and uniform. However, after 1 d of immersion, both coatings showed activation in OCP, owing to the dissolution of splat particles from the corner where the sharp edges are present in the Al coating, while Mg preferentially dissolved in the Al-5 Mg coating and made galvanic cells. Mg is galvanically more active than Al in the Al-5 Mg coating. Due to the capacity of the corrosion products to cover the pores and defects, both coatings stabilized the OCP after 13 d of immersion. The total impedance of the Al-5 Mg coating is gradually increased and is higher than the Al, which can be attributed to the uniform and dense coating morphology where Mg dissolves and agglomerates to form globular corrosion products and deposit over the surface, thereby causing barrier protection. The defect bearing corrosion products on Al coating led to the cause having a higher corrosion rate than the Al-5 Mg coating. A total of 5 wt.% mg in the Al coating improved the corrosion rate by a rate of 1.6 times compared to the pure Al in the 3.5 wt.% NaCl solution after 41 d of immersion.

## 1. Introduction

Steel structures situated near the coastal area are severely corroded, as localized and pitting corrosion is caused by Cl^−^ ions present in the atmosphere. Therefore, sacrificial metals such as Al, Zn, and Mg are being used to reduce the corrosion of steel structures. Different coating systems are being used to deposit the Al, Zn, and Mg coating on the steel surface. Hot dip galvanizing (HDG) is the most popular method to deposit the Zn, Al and Zn-Al, Al-Mg, and Zn-Al-Mg coating. However, this process cannot be used where the steel structures are already corroded due to the limitations of the process. The HDG coating process needs a closed chamber with different complicated steps. The Zn-Al-Mg alloy coating deposited by HDG, meanwhile, is prone to corrosion [1,2,3,4,5,6,7]. Moreover, there are different thermal spray systems where these metallic coatings can be applied on the steel substrate [8,9,10]. In this process, semi-molten metal particles are propelled by the compressed air at a high speed, resulting in the collision of molten particles and the formation of a coating layer.

The thermally sprayed Al coating causes hydrogen embrittlement in steel exposed to alkaline conditions due to over protection [11]. However, Mg provides good protection to the steel [12]. The Al-Mg alloy possesses excellent corrosion resistance in natural, neutral, and alkaline conditions owing to the formation of an insulating layer of Mg^2+^ as well as layered double hydroxides (LDH) on the surface [13,14,15]. The solubility of the Mg in Al is very limited at room temperature, and it can be up to only 5 wt.% in Al. If the amount of Mg is increased any greater than this amount, the solubility of the Mg in the Al matrix leads to brittleness, which is susceptible to pitting corrosion in a saline condition [16,17]. Therefore, 5 wt.% Mg can be soluble in Al-Mg wrought wire casting, and it can be used in a thermal spray coating system.

Due to its ease of use and the accessibility of the spray gun at any location, the arc thermal spray is the method most commonly used to deposit anodic metals on the steel substrate [18]. However, this coating system possesses severe defects/pore formation on the deposited coatings [19,20]. The deposition of different metallic coatings depends on the application used. Alternatively, in the plasma arc thermal spray process, a single metal or alloy wire is used as an anode (consumable), whereas a fixed Cu acts as a cathode (non-consumable). This wire moves with the aid of a wheel to get close to the cathode, where arcing has taken place by creating plasma in normal air at a pressure of three bar. Once the metals or alloy wires have started to melt, the molten metal droplets propelled by the compressed air at six bar result in the deposition of the coating [21,22]. The Al coating is used for wear, erosion, and corrosion resistance, but because an oxide film forms, it is ineffective at giving cathodic protection [23,24,25,26]. The Zn provides cathodic protection to the steel structures in a saline condition due to the galvanically more active galvanic series [22,27]. The Al-Zn coating deposited by the arc thermal spray process exhibits wear and corrosion resistance, Zn provides cathodic protection, and Al erosion resistance [15,28,29,30]. In this coating, Zn preferentially dissolves and forms Zn_5_(OH)_8_Cl_2_ (simonkolleite), Zn_5_(OH)_6_(CO_3_)_2_ (hydrozincite), and Zn-Al layered double hydroxide (Zn_6_Al^2^(OH)_16_CO_3_: Zn–Al LDH) as corrosion products [21]. These corrosion products act as barrier for the penetration of ocean water.

Therefore, in the present study, considering the aforementioned drawbacks of the arc thermal spray process, we deposited Al-5 Mg coating (maximum soluble Mg in Al matrix) by the plasma arc thermal spray process attributed to the excellent mechanical properties and high corrosion resistance in marine application [31,32] when compared with Al coating in 3.5 wt.% NaCl solution until 41 d of immersion. The corrosion kinetics have been explained with the immersion periods. In the present study, the developed plasma arc thermal spray process uses normal gas to create plasma instead of Ar and N_2_ with H and He. The plasma arc thermal spray process is a convenient way to deposit the anodic metals on the steel substrate owing to the high deposition rate, as is the fact that its spray gun is portable, which means that it can brought to any location with other accessories in order to coat the complex and large structures where corrosion has already started.

## 2. Materials and Methods

### 2.1. Materials

A 1.6 mm diameter single wire of Al (99.95 wt.%) and 95 wt.% Al–5 wt.% Mg (Al-5 Mg) was used to deposit 100 μm coating by the plasma arc thermal spray process. The deposition of the coating was performed on 80 mm × 60 mm x 1 mm dimensions of the steel plate. Prior to depositing the coating, the steel substrate was grit blasted by 0.8 mm–1 mm alumina to make the rough substrate. A plasma arc thermal spray system was used to deposit the coating where the spray gun was kept 20–25 cm distance from the steel substrate. The coating was deposited by generating a plasma gas on 65 V and 70 V at 3 bar pressure and 60 mA for the Al and Al-5 Mg coatings, respectively. The Cu (non-consumable) cathode was fixed, whereas the metal to be deposited as an anode (consumable) was moving through the roller. At the intersection where the moving anode reach to the fixed cathode, arcing started, and, with the help of 6 bar compressed air, the molten metal particles propelled and hit the substrate to deposit the coating [21,22].

### 2.2. Methods

The coating thickness of the deposited coatings were performed by a non-destructive Elcometer 456 gauge (Tokyo, Japan) at different places, and the average is mentioned in the manuscript. 

The bond adhesion was measured as suggested in the Korean standard (KS) F4716 [33] at four locations of the deposited coatings. The details of the procedure are explained in our recent publication [21,22].

The surface morphology of coating and corrosion products were performed by scanning electron microscopy (SEM, MIRA3, TESCAN, Brno, Czech Republic) along with elemental composition by energy-dispersive X-ray spectroscopy (EDS). 

The oxides or metals present after the deposition of the coatings were analyzed by X-ray diffraction (XRD, Rigaku, Tokyo, Japan) by Cu Kα radiation at 40 kV and 100 mA.

The contact angle of the deposited coatings was performed by an optical contact angle meter (Smart Drop, Korea) to know the hydrophobicity. 

For the corrosion studies, a 15 mm × 15 mm × 1 mm dimension of steel plate was cut from 80 mm × 60 mm × 1 mm coated samples. The exposed surface area of the coating was 0.78 cm^2^ and it was fixed for each sample. The corrosion studies were performed in duplicate set of samples, and the average is reported in the manuscript. The corrosion experiment was performed in 3.5 wt.% NaCl solution with immersion duration. The electrochemical impedance spectroscopy (EIS, Princeton Applied Research, Oak Ridge, TN, USA) was performed by the three electrode system, where deposited coating works as a working electrode (WE), saturated calomel electrode (SCE) works as a reference electrode, and platinum mesh works as a counter electrode. The EIS was performed by a 10 mV sinusoidal voltage from 100 kHz to 0.01 Hz. Finally, the potentiodynamic polarization (PDP) of the deposited coating after 41 d of immersion in 3.5 wt.% NaCl solution was carried out from −0.40 to 0.80 V versus SCE at a 0.167 mV/s scan rate. The analysis of electrochemical data was performed by Metrohm Autolab Nova 1.10 software (Kanaalweg, Utrecht, The Netherlands).

## 3. Results and Discussion

### 3.1. Coating Thickness and Bond Adhesion

**Figure 1:**
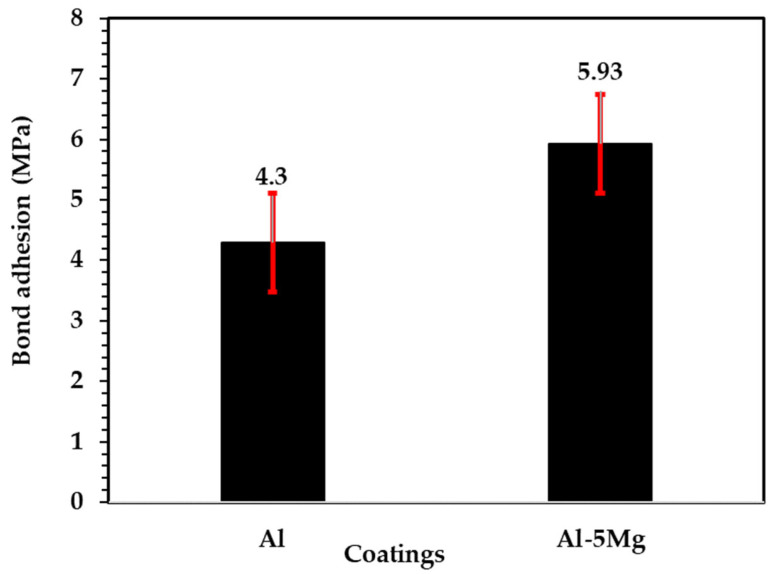
Bond adhesion values of the coatings.

After four passes of the spray gun, the coating thickness of the Al and Al-5 Mg is measured at different locations and is found to be 100 (±10) μm. The coating thickness can be confirmed by cross section SEM images. It will be described in 3.2 SEM of the coatings subsection of the manuscript. The bond adhesion results of the deposited coating are shown in Figure 1. The influence of 5 wt.% Mg in Al can be seen from the bond adhesion results (Figure 1), where it shows a 5.93 MPa adhesion value and is 38% higher than the pure Al coating [34]. This is attributed to the solid solution strengthening during melting and the deposition of the coating on the steel substrate [16,35,36]. The density of the Mg is also lower than Al, where the molten metal particles are entrapped in the mixture and later deposited in the coating, which fills the defects and opens the pores of the Al-5 Mg coating, resulting in a higher bonding strength [37]. 

### 3.2. SEM of the Coatings

**Figure 2:**
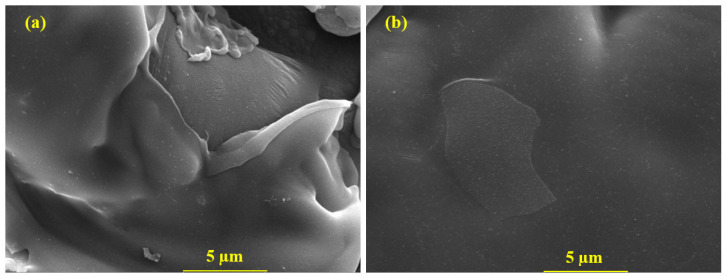
SEM of (**a**) Al and (**b**) Al-5 Mg coatings deposited by plasma arc thermal spray process at 10,000×.

The SEM of the coatings is shown in Figure 2. The Al coating, as shown in Figure 2a, exhibits skin type morphology where the top layer is broken due to the formation of splat on the coating. It is attributed to the high surface tension of the molten Al particles [38] where the metal particles are not be allowed to diffuse in the deposited coating. Therefore, a crack and porosity is developed in the coating. Additionally, the thin splat particles that formed are ascribed to the rapid collision of molten metal droplets [34]. The splat particles’ edges are uplifted due to the high collision of sprayed particles, which are not permitted to firmly attach to the steel substrate and cause internal compressive stress [39]. Therefore, the bond adhesion values of Al coating are lower than the Al-5 Mg coating. According to Figure 2a, the Al coating’s pore size varies from 5 to 10 μm. Alternatively, the Al-5 Mg coating shows a smooth, compact, and uniform layer of the coating (Figure 2b). The Mg in Al-5 Mg coating has a lower density than Al because Mg can melt and be entrapped in a mixture of molten particles, and it can subsequently settle in the defects, filling them and making the coating dense. 

**Figure 3:**
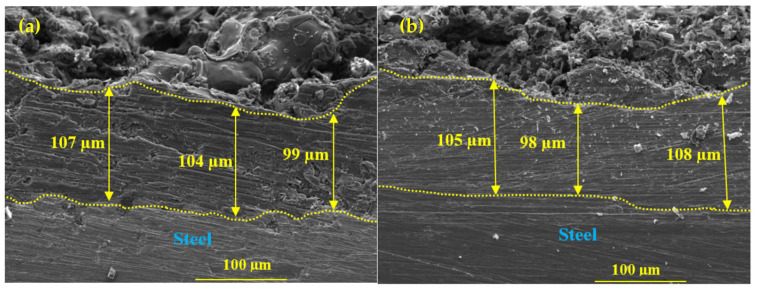
Cross section SEM images of (**a**) Al and (**b**) Al-5 Mg coatings deposited by plasma arc thermal spray process at 500×.

The cross section SEM is shown in Figure 3. The thickness measured by SEM is found to be 100 (±10) μm. The coating thickness measured by Elcometer and SEM are well corroborated. Many pores and defects can be seen in Figure 3a, mostly at the interface of the coating/steel substrate. It may be that during the first pass of the spray gun to deposit the coating, the Al is not melted properly due to having less time to melt the wires; therefore, splat is observed in Figure 2a. On the other hand, the influence of Mg can be seen from the cross section SEM of Al-5 Mg coating in Figure 3b. This coating become dense and few defects or pores are observed. This is ascribed to Mg, which deposits in coating pores/defects [38] due to low density. Therefore, the porosity of the coatings considering the cross section of the SEM is measured. The porosity of the coating is measured by open source ImageJ software (version 1.52n) (NIH, Bethesda, MD, USA) [40,41,42,43]. The porosity is found to be 10.86% and 2.49% for Al and Al-5 Mg coatings (Table 1), respectively. The porosity of the Al-5 Mg coating is reduced by more than four times compared to the Al coating. A total of 5 wt.% Mg in Al enhanced the physical properties of the deposited coating.

The EDS analysis of the deposited coating is shown in Table 1. The oxygen (O) content in Al coating is found to be 2.63 wt.% e.g., slightly greater than Al-5 Mg. This oxygen might be coming from the atmosphere or inflight particles during the deposition of the coatings. Moreover, the Mg content in Al-5 Mg coating is found to be 4.76 wt.%, and it is almost equivalent to the started materials, e.g., the feed stock wire used to deposit the coating.

### 3.3. XRD of the Coatings

**Figure 4:**
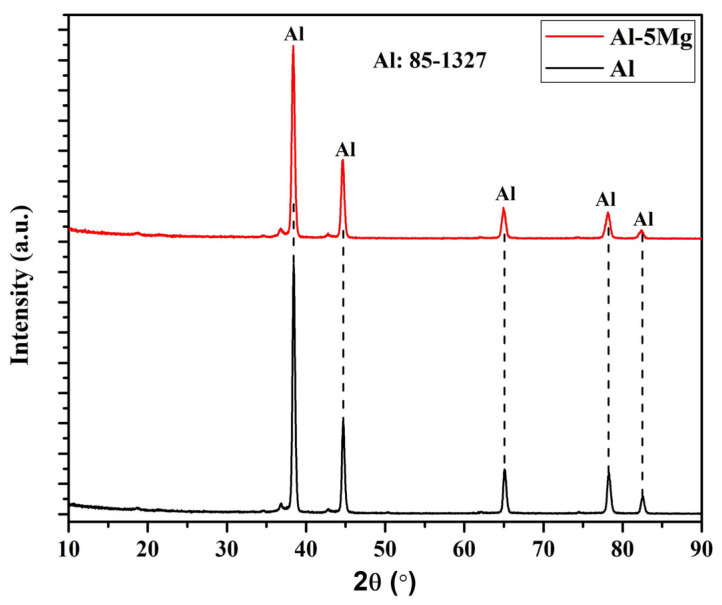
XRD of the coatings.

The XRD of both coatings exhibit only the Al phase (JCPDF: 85-1327), as shown in Figure 4. There is no peak of Mg in the Al-5 Mg coating, and oxides in both coatings [15] might be limitation of XRD, which could not detect a low amount or a very thin layer of oxide film. This result corroborates the EDS analysis, where a lower amount of oxygen is observed and forms oxides to be detected by XRD. The peak intensity of Al in the Al-5 Mg coating is lower than pure Al, showing the effect of Mg. There is another possibility, which is that, generally, the thermal spray coating system enables mechanical bonding with the substrate rather than chemical, and this does not allow the formation of intermetallic as observed in hot dip galvanization. Therefore, intermetallic phases are absent.

### 3.4. Contact Angle of the Coatings

**Figure 5:**
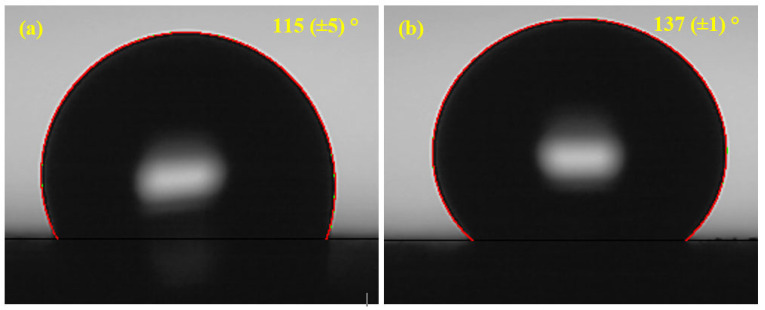
Contact angle of (**a**) Al and (**b**) Al-5 Mg coatings.

The water contact angle with the coating is shown in Figure 5. Both the coatings show hydrophobicity where contact angle is greater than 90°. However, the contact angle of Al and Al-5 Mg coatings exhibits at 115 (±5)° and 137 (±1)°, as shown in Figure 5a,b, respectively. The lower contact angle of the Al coating can be attributed to the presence of defects and pores, which allows the water molecule to ingress. The high surface roughness or defects decrease the hydrophobicity, too [44]. This implies that adding 5% Mg to Al improves its water repellent properties. This result suggests that Al-5 Mg coating could exhibit lower roughness and provide greater corrosion resistance. This result is well corroborated with the SEM of the coatings where the Al coating possesses greater porosity and defects.

### 3.5. Corrosion Studies in 3.5 wt.% NaCl at Different Duration of Immersion

#### 3.5.1. Open Circuit Potential (OCP) Measurements

**Figure 6:**
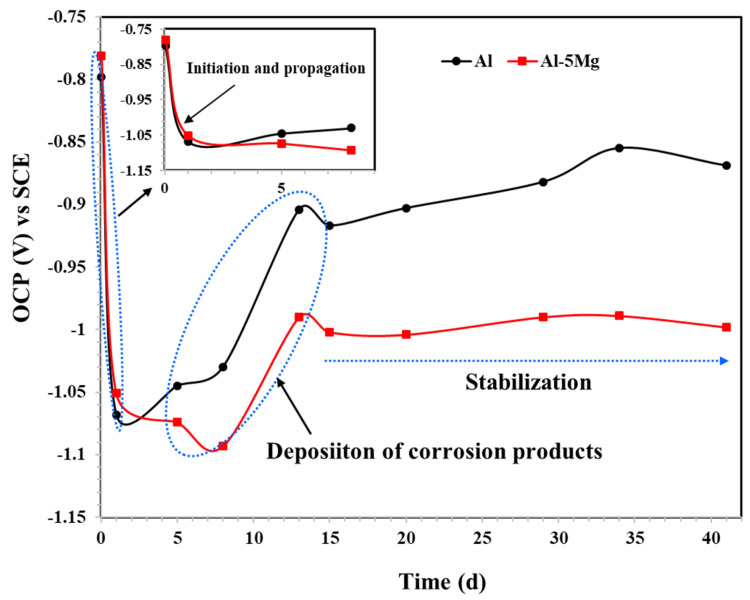
OCP of plasma arc thermal sprayed coating in 3.5 wt.% NaCl solution.

The corrosion characteristics of the Al and Al-5 Mg coating is assessed by OCP measurement with immersion duration, and the results are shown in Figure 6. During initial periods of immersion, both coatings exhibited electropositive OCP attributed to the formation of native oxide [45,46,47] and yet not observed in XRD on the Al coating, while Al-5 Mg coating exhibited uniform morphology and a high contact angle that does not allow the solution to ingress into the coating. This result is corroborated by other authors [48,49]. However, once the duration is increased, there is a possibility of native oxides in the Al coating, whereas in the case of the Al-5 Mg coating, the Mg preferentially dissolve and initiate the corrosion reaction, i.e., the initiation process, as shown in the inset of Figure 6. The natural passivation film on the Al coating depends on the surface condition [50] in the present study, where defects and pores are formed, which leads to the severe dissolution of the coating. Therefore, from 1 h to 41 d of immersion, the corrosion process is categorized in three stages––i.e., initiation and propagation of the corrosion reaction, deposition of the corrosion products, and the stabilization of the corrosion reaction. From 1 h to 24 h (1 d) of immersion, the OCP of the Al coating shifted from −0.798 to −1.068 V vs. SCE, whereas the Al-5 Mg coating did so from −0.781 to −1.051 V vs. SCE. Meanwhile, during the active dissolution of coatings, some oxides are used to form and fill/deposit into the defects of the coating where the OCP again started to shift towards an electropositive direction, which shows the passive film formation. These periods (the second stage), i.e., from 5 d to 13 d, are considered as the deposition of the corrosion products and the oxide film. From 13 d to 41 d of immersion, the Al-5 Mg coating exhibits active OCP compared to the Al, which is due to the galvanic cell as well as Mg having more electronegative standard potential (Mg = −2.380 V vs. standard hydrogen electrode (SHE) at 25 °C). This means that Mg preferentially dissolves in the Al-5 Mg coating. Once the immersion duration is extended from 13 d to 41 d, the OCP of both coatings is stabilized. This step is called the stabilization of the OCP, where the corrosion reaction of the coatings is diminished [49,51]. However, in both coatings, the Al-5 Mg coating exhibited greater cathodic protection whereas the OCP remained constant at −0.998 V vs. SCE from 13 d to 41 d, and in the case of Al coating, meanwhile, the fluctuation is observed from around −0.900 to −0.870 V vs. SCE due to the pits formation in the chloride laden solution. This means that Al exhibits its OCP at borderline, where the minimum OCP should be −0.870 V vs. Ag/AgCl for the cathodic protection [34].

#### 3.5.2. Electrochemical Impedance Spectroscopy (EIS) with Immersion Duration

**Figure 7:**
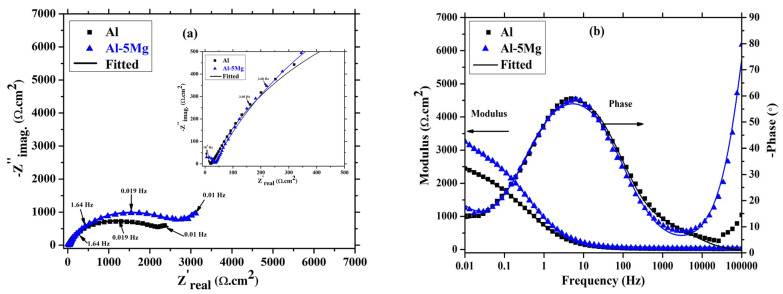
EIS (**a**) Nyquist and (**b**) Bode plots of coatings after 1 h of immersion in 3.5 wt.% NaCl solution.

The EIS of the coatings for 1 h of immersion is depicted in Figure 7. The coatings immersed in 3.5 wt.% NaCl solution initiate the dissolution, but due to the very limited time of immersion, it shows that it is three times constant in Nyquist plots (Figure 7a) at high, middle, and low frequency. It can be seen from Figure 7a that the trend in Nyquist plots for the Al and the Al-5 Mg coatings is identical, and can be attributed to the coating characteristics. Moreover, the Nyquist plot magnitude of the Al-5 Mg coating is greater than pure Al. This result suggests that the Al-5 Mg coating exhibits higher corrosion resistance than Al. There is a distinct capacitive loop at high frequency (inset of Figure 7a) observed in the Al-5 Mg coating, revealing the formation of oxide/barrier film or dense morphology of the coating, which cause barrier. This result is well corroborated with SEM (Figure 2b and Figure 3b) where Al-5 Mg coating shows dense and uniform surface. There is no significant difference in the middle frequency capacitive loop of both coatings. However, the low frequency capacitive loop of Al-5 Mg coating is greater than Al caused by charge transfer resistance (*R_ct_*). 

The Bode plots of coatings after 1 h of immersion is shown in Figure 7b. The total impedance of the Al-5 Mg coating at 0.01 Hz is about 1.5 times greater than that of the Al coating. It means Al-5 Mg coating has a higher corrosion resistance than the Al coating after 1 h of immersion in 3.5 wt.% NaCl solution. The phase-frequency Bode plots of both coatings at high frequency reveal the oxide film formation, as has been suggested by other authors [52,53,54,55,56]. The Al-5 Mg coating exhibits around a −80° phase angle maxima at 100 kHz due to the surface properties where this coating shows dense and uniform morphology as well as a high contact angle. When the coating is immerged in NaCl solution, the active centers (defects/pores) in the coating initiate the corrosion reaction and form corrosion products, which causes barrier kinds of protection. In the case of the Al coating, the high frequency phase angle maxima are lower than the Al-5 Mg, which shows the formation of loose or porous corrosion products. The middle frequency capacitive loop of both coatings exhibited around −60°, which inferred the capacitive nature owing to the nature of the coating. The low frequency phase angle maxima of the Al-5 Mg coating are greater than the Al, which shows the high *R_ct_* value where the cathodic reaction is controlled.

**Figure 8:**
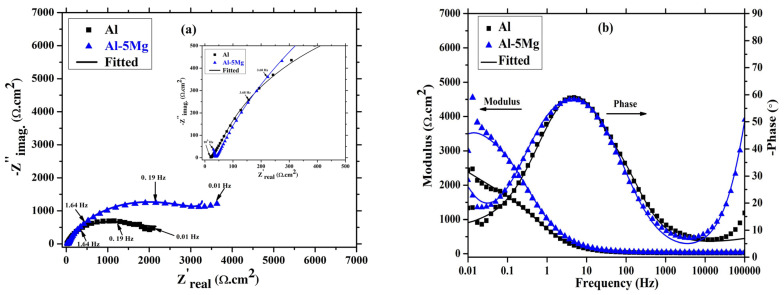
EIS (**a**) Nyquist and (**b**) Bode plots of coatings after 8 d of immersion in 3.5 wt.% NaCl solution.

The corrosion products/oxides are formed during the dissolution of a metallic coating immersed in a NaCl solution and filling the active centers. Therefore, the corrosion resistance properties of the coatings are increased. In the present study, after 8 d of immersion in 3.5 wt.% NaCl, the EIS results are depicted in Figure 8. Moreover, in the case of the Al coating, the magnitude of the Nyquist plots after 8 d of immersion (Figure 8a) are identical, as observed after 1 h, which shows the retaining corrosion resistance properties. In this case, there are many factors involved, unless electrochemical parameters are not calculated. The coating resistance (*R_c_*) of the Al is decreased after 8 d compared to 1 h, as shown in Table 2, but, in the meantime, the oxide film resistance (*R_ox_*) and charge transfer resistance (*R_ct_*) are increased (Table 2). Therefore, the Al coating exhibits identical corrosion resistance properties after 8 d as that observed for 1 h. In the case of the Al-5 Mg coating, the Nyquist plot magnitude is increased after 8 d, as observed in Figure 8a, compared to 1 h. This result suggests that the Mg in the Al-5 Mg coating makes the coating galvanically active rather than the surface morphology, which enhances the dissolution of the coating, and, therefore, the OCP is shifted towards active direction (Figure 6) and whatever the corrosion products/passive film are formed deposit over the coating surface and make it immune. Thus, the total impedance of the Al-5 Mg coating at 0.01 Hz is increased (Figure 8b) by 1.5 times than 1 h. The impedance is mostly increased at a low studied frequency, suggesting *R_ct_* where the cathodic reaction is slowed down [57]. There is a shift in the high frequency phase angle maxima towards a lower angle of Al-5 Mg coating, as shown in Figure 8b, which shows the dissolution of the coating due to the presence of the Mg, which is galvanically more active than the Al. On the other hand, the low frequency capacitive loop/phase angle maxima are shifted towards a higher angle, suggesting that the majority of the corrosion resistance is caused by *R_ct_*. Alternatively, the middle frequency phase angle maxima for both coatings is identical, as observed after 1 h, but in the case of the Al-5 Mg coating, a broadening is observed, which reveals the enhanced corrosion resistance properties.

**Figure 9:**
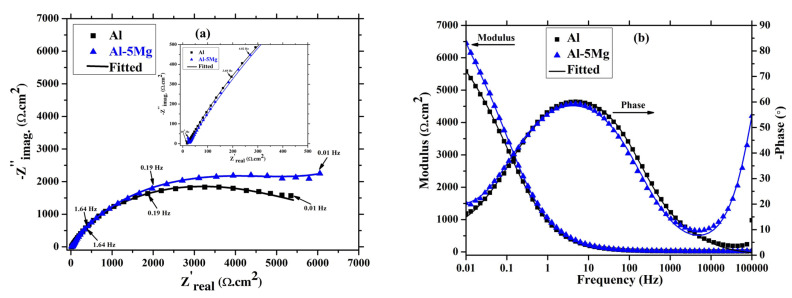
EIS (**a**) Nyquist and (**b**) Bode plots of coatings after 41 d of immersion in 3.5 wt.% NaCl solution.

The coatings are immersed in 3.5 wt.% NaCl solution for extended periods, and their corrosion resistance properties are assessed. The magnitude of the Nyquist plots for both coatings are increased after 41 d of immersion, as shown in Figure 9a, compared to earlier immersion periods. This is attributable to the filling of the pores/defects of the coatings by the corrosion products, which reduce the active centers and enhance the corrosion resistance. The Nyquist plots magnitude of the Al coating is increased to a still greater extend compared to earlier immersion periods, and this is attributable to the more active centers (pores/defects) in the coating, which started to dissolve after 8 d and made a passive layer that resulted in a barrier on the coating surface. The high and middle frequency capacitive loops of both coatings are smaller in size compared to the low frequency, as shown in the inset of Figure 9a, which suggests that the corrosion protection provided by *R_ct_* caused at coating/steel interface, where some oxides are formed and inhibit the transfer of electrons from the steel substrate [57].

The total impedance of Al and Al-5 Mg coatings after 41 d of immersion in 3.5 wt.% NaCl solution are increased by 2 and 1.5 times (as shown in Figure 9b) compared to 8 d, respectively. The increment in total impedance of the Al coating is greater than the Al-5 Mg. Up to 8 d of immersion, there is no significant change in total impedance of Al coating, but once the duration is extended, the dissolution of the Al is greater. As a result, *R_c_* is decreased (Table 2), and, moreover, the deposition of the corrosion products is also increased, which means that the intensity of the increment in total impedance is greater than the Al-5 Mg coating. Moreover, the total impedance of the Al-5 Mg coating is higher than the pure Al coating. This finding suggests that the corrosion products/oxide films formed on the Al coating are porous and defective, which allow the solution to penetrate towards the steel substrate. 

The phase-frequency Bode plots of the coatings after 41 d of immersion are shown in Figure 9b. The high frequency capacitive loop at −55° still exists in the Al-5 Mg coating after 41 d, as was also observed for 8 d. This means that the corrosion protection provided by this coating is attributable to the surface morphology, which acts as a barrier. The phase angle maxima of both coatings at the middle and low frequencies remains unchanged, revealing that whatever corrosion reaction has occurred after 8 d of immersion, an identical phenomenon is then also observed after 41 d. This finding suggests that both coatings provide corrosion protection at a longer duration, but that the Al-5 Mg one does so to a greater extent. Initially, the Al coating deteriorated due to the presence of defects/pores, while the Al-5 Mg coating led to corrosion because of the Mg, which is galvanically more active than the Al. 

**Figure 10:**
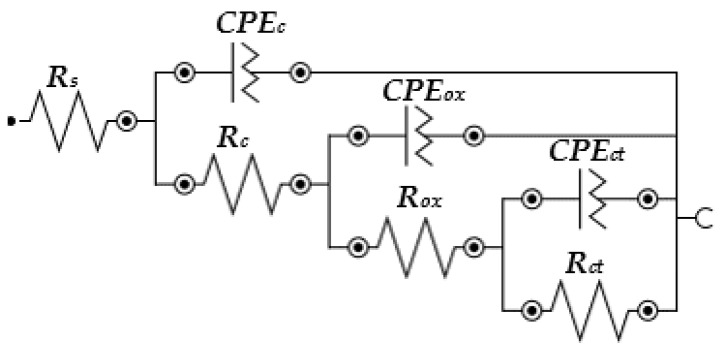
Schematic of EEC for the coatings.

The EIS data are fitted in a suitable electrical equivalent circuit (EEC) and the fitted data overlap with the raw data, as shown Figure 7, Figure 8 and Figure 9. The EEC fitted for the Al and the Al-5 Mg coating at different durations of immersion is shown in Figure 10. In this circuit, three times constants are involved at high, middle, and low frequencies. The first and second time constants are caused by resistance and the capacitance as constant phase element (CPE) for coating (*CPE_c_*) and oxide/passive film (*CPE_ox_*), respectively, while the third time constant is for charge transfer resistance (*R_ct_*) [58,59]. In this EEC, *R_s_* related to solution resistance, and *CPE_c_* and *R_c_* for coating. The CPE is observed instead of the pure capacitance due to the surface heterogeneity. There are oxide/passive films formed once the coating is immersed in the solution, and, therefore, the constant phase element for the oxide film (*CPE_ox_*) and oxide film resistance (*R_ox_*) are fitted. Moreover, the solution reached at the coating/steel interface that caused the charge transfer resistance (*R_ct_*) and the constant phase element for charge transfer (*CPE_ct_*).

The electrochemical parameters after the fitting of the EIS plots in EEC is shown in Table 2. The *R_s_* values of both coatings are found from 5–10 Ω·cm^2^ at different durations of immersion. The *R_c_* of the Al coating is gradually decreased, suggesting the dissolution caused by pores/defects present in the coating where *n_c_* (*CPE_c_* exponent of the coating) and *Q_c_* (CPE coefficient of the coating) values are gradually decreasing and increasing with the immersion time, respectively. The high value of *Q_c_* indicates the capacitive properties of the coating owing to the presence of defects. On the other hand, there is no change in *R_c_* of the Al-5 Mg coating from initial to extended periods of immersion. This result suggests that the Al-5 Mg coating is uniform and dense. However, due to the presence of Mg in the Al-5 Mg coating, it corrodes, but, in the meantime, it could form a passive film, and, thus, the *R_ox_* values are increased with time and *R_c_* remains constant. Moreover, after 41 d of immersion, the *R_ox_* values of Al coating increased by 2.5 times compared to 8 d. This means that until 8 d of immersion, the coatings deteriorate and then the corrosion products deposit onto the coating surface, and the *R_ox_* is thereby increased. The low *n_ox_* (CPE exponent of oxide film) and high *Q_ox_* (CPE coefficient of oxide film) values indicate that the corrosion products are porous, whereas in the case of Al-5 Mg, they are vice versa, which suggests that oxide films are homogenous and less defective. Therefore, the *R_ox_* is greater than the Al coating. This also leads to an increase in the *R_ct_* value at the same time. On the contrary, the *R_ct_* of the Al-5 Mg coating gradually increases with immersion time, which suggests that it was the formation of the stable layer of oxides at the coating/steel interface that led to an increase in the corrosion resistance. The *n_ct_* (CPE exponent of charge transfer) values of the Al-5 Mg coating from the initial period to 41 d of immersion is greater than 0.80, which suggests that the homogenous nature of the corrosion products/passive film formed at the coating/steel interface. In the case of the Al coating, this value is also ≥0.8, which indicates the homogenous nature of the corrosion products, although it is less than the Al-5 Mg coating.

#### 3.5.3. Potentiodynamic Polarization (PDP) Analysis

**Figure 11:**
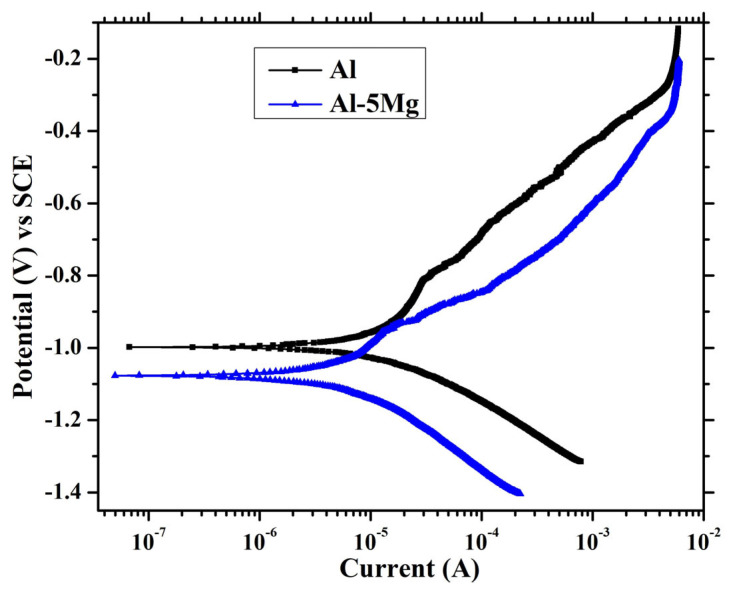
PDP plots of coatings after 41 d of immersion in 3.5 wt.% NaCl solution.

The PDP results of both coatings after 41 d of immersion in 3.5 wt.% NaCl solution are depicted in Figure 11. The Al and Al-5 Mg coatings are cathodically polarized, and this can be attributed to the oxygen-reduction reaction where the oxide film at the coating/steel interface is reduced. The cathodic current density of the Al is greater than the Al-5 Mg coating, which suggests the formation of loose or unstable oxide film, although it can easily be reduced during cathodic scanning. On the other hand, the Al-5 Mg coating is cathodically more polarized than the Al. On the contrary, considering the anodic current density, the Al-5 Mg coating is electrochemically more active, which is attributable to the dissolution-deposition mechanism [60], where the Mg leads to the dissolution of the coating but at the same time deposits oxide film on the coating surface. 

The electrochemical parameters are extracted after fitting the PDP curve in Tafel regions, and the corrosion rate (μm/year) is calculated according to ASTM G102 [61].

(1) $$\mathrm{Corrosion}\;\mathrm{rate}\;({\mu m\cdot \mathrm{year}}^{-1})=\frac{3.27\times i_{corr}\times E.W.}{d}$$

In Equation (1), *i_corr_*, E.W., and d equal the corrosion current density and the equivalent weight and density, respectively. The fitted results are shown in Table 3. The corrosion potential (*E_corr_*) of the Al-5 Mg coating is 100 mV more active than the Al, which is attributable to the presence of Mg in the coating. However, the *i_corr_* of the Al and the Al-5 Mg coatings are found to be 3.83 and 2.35 μA·cm^−2^, respectively. The reduced *i_corr_* value of the Al-5 Mg coating suggests that Mg has the beneficial effect of mitigating the corrosion. The corrosion rate of the Al and the Al-5 Mg coating is found to be 41.75 and 26.56 μA·cm^−2^. The Al coating is more susceptible to corrosion in 3.5 wt.% NaCl solution than the Al-5 Mg coating when deposited by the plasma arc thermal spray process.

### 3.6. SEM of the Corrosion Products

**Figure 12:**
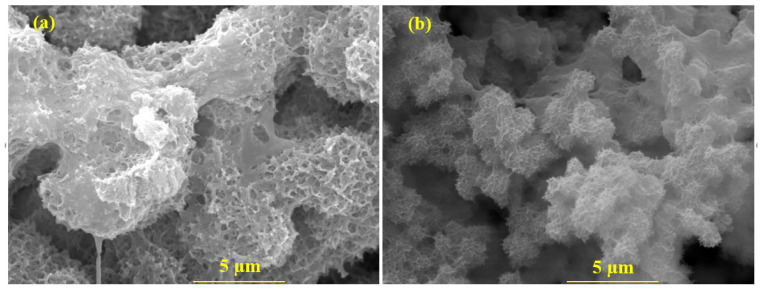
SEM of corrosion products formed on (**a**) Al and (**b**) Al-5 Mg coatings after 41 d of immersion in 3.5 wt.% NaCl solution.

The SEM of the corrosion products after 41 d of immersion in 3.5 wt.% NaCl solution are shown in Figure 12. The corrosion products’ morphology of Al coating is coagulated, as shown in Figure 12a. It can also be seen from Figure 12a that most of the dissolution of the coating started at the corner of the splat particles that were formed after the deposition of the coating (see Figure 2a), and that led to the center. Therefore, a sunken morphology can be observed at every corner along with the multilayer corrosion products (Figure 12a). However, the corrosion products consist of micro/nano pores and uneven morphology. Even after 41 d of immersion, the corrosion products are defective where the Cl^−^ and H^+^ ions are accumulated in the pits [62,63,64], and, therefore, exhibit a high cathodic current density, *i_corr_*, and corrosion rate (Table 3). The Mg in the Al-5 Mg coating makes the surface active for dissolution, and, as a result, uniform corrosion can be observed (Figure 12b) and the corrosion products laminate the coating surface. In this case, the Mg contributes to super saturation and leads to solid solution strengthening as well as possibly being formed by β-phase, which is deleterious in nature [32,65,66,67,68], and, as a consequence, pits are observed in Figure 12b. The globular corrosion products help to fill the active centers of coating, which makes them dense and leads to the corrosion protection. The particle size of the globular corrosion products is less than 1 μm, which combine with each other and fill the pits of the coating caused during the immersion periods. Therefore, the globular size corrosion products help to improve the corrosion resistance of the Al-5 Mg coating.

The EDS analysis of the corrosion products are shown in Table 4, where it can be seen that the corrosion products formed on the Al coating exhibit significant reduction in amount of Al, even though it is lower than oxygen. This result suggests that most of the Al is corroded and might form Al(OH)_3_/Al_2_O_3_ after 41 d of immersion in 3.5 wt.% NaCl solution. There is a negligible amount of Na and Cl observed, though, which suggests that the corrosion reaction occurred via an oxygen-reduction reaction. On the contrary, in the case of the Al-5 Mg, Na and Cl is seen in greater amounts than the pure Al coating, which suggests that NaCl might deposit in the pits of the coating due to the selective anodic dissolution of Mg. Hence, only 1.27 wt.% Mg remained in the coating.

## 4. Conclusions

Two different types of coatings, i.e., Al and Al-5 Mg, are deposited by the plasma arc thermal spray process, and their corrosion characteristics are determined in 3.5 wt.% NaCl solution until 41 d of immersion. The Al coating exhibited splat particles with sharp edges at the corner as well as greater porosity than the Al-5 Mg coating. On the other hand, the Al-5 Mg coating exhibited dense and uniform morphology. As a result, the porosity is decreased by four times when compared to the Al coating, resulting in 1.4 times better bond adhesion values. This is attributable to the solid solution strengthening during melting and the deposition of the coating on the steel substrate. Initially, both coatings exhibited electropositive OCP that can be attributed to both the formation of native oxide in the Al coating and the preferential dissolution of Mg (galvanically more active than Al) in the Al-5 Mg coating. In the meantime, the dissolved oxides/coatings fill out the defects, and, therefore, after 13 d of immersion, the OCP is stabilized due to the pores/defects filling activity by the corrosion products. The Al coating from the initial periods until 41 d of immersion observed a dissolution where the *R_c_* value is gradually decreased. However, in the case of the Al-5 Mg coating, the corrosion protection is mostly provided by coating as well as *R_ct_*, where its values are significantly increased with time, which is attributable to the formation of globular and dense corrosion products morphology caused by Mg. Therefore, the Al-5 Mg coating exhibited a reduced corrosion rate compared to pure Al coating, plus the presence of pits in the corrosion products of Al-5 Mg coating due to the selective dissolution of Mg.

## Figures and Tables

**Table 1:** EDS analysis of the deposited coatings.

Coatings	Elements (wt.%)			Porosity (%)
	Al	Mg	O	
Al	97.37	-	2.63	10.86
Al-5 Mg	92.98	4.76	2.26	2.49

**Table 2:** Electrochemical parameters of the coatings.

Coatings	Parameters		1 h	8 d	41 d
**Al**	*R_s_* (Ω·cm^2^)		9.62	8.76	6.82
	*R_c_* (Ω·cm^2^)		680.21	653.36	547.59
	*CPE_c_*	*Q_c_* (1 × 10^−5^) (Ω^−1^·cm^−2^·s^−n^)	11.23	11.40	13.65
		*n_c_*	0.74	0.74	0.73
	*R_ox_* (Ω·cm^2^)		730.46	737.62	1862.01
	*CPE_ox_*	*Q_ox_* (1 × 10^−5^) (Ω^−1^·cm^−2^·s^−n^)	10.02	9.96	7.20
		*n_ox_*	0.77	0.77	0.79
	*R_ct_* (Ω·cm^2^)		1016.10	1043.23	3175.04
	*CPE_ct_*	*Q_ct_*(1 × 10^−5^) (Ω^−1^·cm^−2^·s^−n^)	8.81	8.51	5.23
		*n_ct_*	0.80	0.80	0.83
**Al-5 Mg**	*R_s_* (Ω·cm^2^)		7.09	5.13	6.47
	*R_c_* (Ω·cm^2^)		920.17	912.17	906.16
	*CPE_c_*	*Q_c_* (1 × 10^−5^) (Ω^−1^·cm^−2^·s^−n^)	9.88	9.91	9.95
		*n_c_*	0.79	0.79	0.79
	*R_ox_* (Ω·cm^2^)		1033.40	1592.33	2063.76
	*CPE_ox_*	*Q_ox_* (1 × 10^−5^) (Ω^−1^·cm^−2^·s^−n^)	8.51	7.10	5.83
		*n_ox_*	0.82	0.83	0.84
	*R_ct_* (Ω·cm^2^)		1307.10	1991.74	3501.30
	*CPE_ct_*	*Q_ct_* (1 × 10^−5^) (Ω^−1^·cm^−2^·s^−n^)	6.79	5.72	3.38
		*n_ct_*	0.84	0.85	0.87

**Table 3:** Electrochemical parameters of the coating extracted after fitting in Tafel regions.

Coatings	Electrochemical Parameters		
	*E_corr_* (V) vs. SCE	*i_corr_* (μA·cm^−2^)	Corrosion Rate (μm·Year^−1^)
Al	−0.99	3.83	41.75
Al-5 Mg	−1.09	2.35	26.56

**Table 4:** EDS analysis of the corrosion products.

Coatings	Element (wt.%)				
	Al	Mg	O	Na	Cl
**Al**	46.85	-	52.95	0.00	0.20
**Al-5 Mg**	67.99	1.27	28.64	1.45	0.65

## Data Availability

Data will be made available on request.
